# Vitamin D and diabetes mellitus

**Published:** 2014

**Authors:** Fevzi Nuri Aydin, Ibrahim Aydin, Mehmet Aydin

**Affiliations:** 1 Department of Biochemistry, Sirnak Military Hospital, Sirnak, Turkey.; 2 Department of Biochemistry, Sarikamis Military Hospital, Sarikamis, Kars, Turkey.; 3 Department of Biochemistry, Agri Military Hospital, Agri, Turkey.

We read with great interest the article of Bayani et al. on the “Vitamin-D status in diabetic patients” published in Caspian Journal of Internal Medicine (CJIM) (1). Bayani et al. provided a timely and useful summary of the status of vitamin D (vit D) deficiency in type 2 diabetes mellitus. They reported some interesting results based mostly on statistical associations that vit D concentration was significantly lower in type 2 diabetic patients than the healthy individuals. However, we think that some points should be discussed.

The study reported the prevalence of risk factor for both the lowest quartile (<21 ng/mL) and the highest quartile (≥31 ng/mL) of serum vit D levels in the study population. Women, Iranian people and older people were most likely to have low vit D levels. Several studies showed that two previous decades displayed a high prevalence of vit D deficiency in tropical countries such as China, Turkey, India, Iran and Saudi Arabia and estimated its prevalence between 30% and 93% (2, 3). These factors could have affected the results of the study. The frequency of female patients wearing covered dresses should have been mentioned. UV-B radiation does not penetrate glass, so exposure to sunshine indoors through a window does not produce vit D (3). The environmental conditions of the subjects in this study should be presented. Older adults have a reduced level of 7-dehydrocholesterol, so they cannot synthesize vit D as well. Also, their kidneys are less able to produce the active hormone, 1,25 dihydro vit D (3, 4). The mean age of the case group was 51.2±7.98 and in control group was 50.6±7.73 years in this study. Therefore, all the subjects’ kidney and liver function tests should be evaluated. 

Melanin in the darker skin reduces the ability to make vit D in response to sunlight exposure, because it absorbs the sunlight (2, 5). The frequency of dark skinned subjects should be mentioned in the study. Obesity was not in exclusion criteria of the study. Vit D is fat-soluble; it is sequestered in the body fat not allowing it to circulate. Also, those who have had a gastric bypass do not have a portion of their small intestine and cannot readily absorb vit D (6). In conclusion, it would be appreciated if the authors could present some more data adjusted for major confounders including subjects’ kidney and liver function tests, obesity, sunlight exposure and the frequency of female patients wearing covered dresses. This could provide the readers of the journal clearer information in relation to the vitamin D link in patients with type-2 diabetes mellitus.
